# *TaBAS1* encoding a typical 2-Cys peroxiredoxin enhances salt tolerance in wheat

**DOI:** 10.3389/fpls.2023.1152375

**Published:** 2023-03-14

**Authors:** Guilian Xiao, Mingming Zhao, Qinghua Liu, Junzhi Zhou, Zhaohui Cheng, Qiannan Wang, Guangmin Xia, Mengcheng Wang

**Affiliations:** The Key Laboratory of Plant Development and Environment Adaptation Biology, Ministry of Education, School of Life Science, Shandong University, Qingdao, China

**Keywords:** wheat, salt, peroxiredoxin, ROS, yield

## Abstract

Efficient antioxidant enzymatic system contributes to salt tolerance of plants *via* avoiding ROS over-accumulation. Peroxiredoxins are crucial components of the reactive oxygen species (ROS) scavenging machinery in plant cells, but whether they offer salt tolerance with potential for germplasm improvement has not been well addressed in wheat. In this work, we confirmed the role of a wheat 2-Cys peroxiredoxin gene *TaBAS1* that was identified through the proteomic analysis. *TaBAS1* overexpression enhanced the salt tolerance of wheat at both germination and seedling stages. *TaBAS1* overexpression enhanced the tolerance to oxidative stress, promoted the activities of ROS scavenging enzymes, and reduced ROS accumulation under salt stress. *TaBAS1* overexpression promoted the activity of ROS production associated NADPH oxidase, and the inhibition of NADPH oxidase activity abolished the role of *TaBAS1* in salt and oxidative tolerance. Moreover, the inhibition of NADPH-thioredoxin reductase C activity erased the performance of *TaBAS1* in the tolerance to salt and oxidative stress. The ectopic expression of *TaBAS1* in Arabidopsis exhibited the same performance, showing the conserved role of 2-Cys peroxiredoxins in salt tolerance in plants. *TaBAS1* overexpression enhanced the grain yield of wheat under salt stress but not the control condition, not imposing the trade-offs between yield and tolerance. Thus, *TaBAS1* could be used for molecular breeding of wheat with superior salt tolerance.

## Introduction

High salinity is one of the major environmental constraints on plant growth and crop production. To identify salt tolerant genes not imposing the trade-offs between salt tolerance and growth/yield is crucial for building the germplasms to confront the circumstance of salt stress (Laitinen and Nikoloski, 2022). As a typical physiological response to salt and other abiotic stresses, reactive oxygen species (ROS) often over-accumulate in plants, which results in serious secondary damage in cells (Choudhury et al., 2017). Thus, the genes efficiently avoiding ROS over-accumulation would be the excellent candidates for molecular breeding (Choudhury et al., 2017).

Plants possess a complicated ROS scavenging system, including a set of enzymes such as superoxide dismutase (SOD), catalase (CAT), and ascorbate peroxidase (APX) (Mittler, 2002). Besides, peroxiredoxins (Prxs) are simple proteins with peroxidase activity on a variety of peroxide substrates, such as hydrogen peroxide (H_2_O_2_) (Vidigal et al., 2015). They compromise several isoforms that are specifically distributed in cytosol, chloroplasts as well as other organelles and even in the extracellular space (Meyer et al., 2008). Prxs are characterized by a peroxidatic cysteine (Cys) at the active site, which could be oxidized by the peroxide substrates to reduce the peroxides to the corresponding hydroxylated compounds (Puerto-Galán et al., 2013). According to the peroxidatic cysteine, Prxs are differentiated into two categories, 1-Cys and 2-Cys Prxs (Puerto-Galán et al., 2013). 2-Cys Prxs are homodimeric enzymes where the two subunits are linked covalently *via* a disulphide bridge in the oxidized form (Puerto-Galán et al., 2013; Cerveau et al., 2016). 2-Cys Prxs highly accumulate in the chloroplasts, and they contain an N-terminal extension which is important for importing the preprotein into the chloroplasts (Dietz, 2011). The chloroplast is not only the site of photosynthesis, but also an important source of H_2_O_2_. Abiotic stresses have been found to induce ROS over-production in the chloroplasts (Li and Kim, 2021). 2-Cys Prxs have proved to play the central role of in chloroplast redox regulation (Ojeda et al., 2018), and they participate in the response to oxidative and abiotic stresses such as high temperature through modulating chloroplast redox (König et al., 2002; Vidigal et al., 2015; Li et al., 2016; Mishra et al., 2021). However, whether they offer the tolerance to salt, a major abiotic stress crops confront, has not been reported.

Upon ROS, the peroxidatic Cys of 2-Cys Prxs becomes transiently oxidized as sulfenic acid (-SOH) and then condenses with the resolving Cys to form a disulfide bridge, and this disulfide has to be reduced for a new catalytic cycle (Puerto-Galán et al., 2013). The chloroplast localized NADPH-dependent thioredoxin reductase C (NTRC) is an efficient reductant of 2-Cys Prxs (Moon et al., 2006; Pérez-Ruiz et al., 2006; Alkhalfioui et al., 2007; Ojeda et al., 2018), indicating a possible relationship between antioxidant mechanisms and redox regulation. NTRC participates in the redox regulation of chloroplast enzymes that depend on 2-type Thioredoxins (Trxs) through the control of the redox balance of 2-Cys Prxs (Pérez-Ruiz et al., 2017). The redox status of 2-Cys Prxs was severely impaired in the *ntrc* mutant (Pulido et al., 2010). The association between 2-Cys Prxs and NTRC in the response of salt and other abiotic stresses has not been reported.

Besides as toxic molecules, ROS serve as the signals that are crucial for regulating diverse biological processes (Mittler et al., 2011; Suzuki et al., 2012; Sierla et al., 2013). Thus, nontoxic ROS levels need to be fine-tuned to maintain suitable homeostasis *via* the orchestration by both ROS production machinery involving ROS-producing enzymes and the metabolic counter-process involving ROS-scavenging enzymes (Miller et al., 2009). NADPH oxidases (NOX) are the major producers of ROS (Torres and Dangl, 2005; Suzuki et al., 2011). The inhibition of NOX by mutating the encoding gene or applying the NOX inhibitors reduces ROS production, abiotic stress tolerance, and antioxidant defense capacity (Torres and Dangl, 2005; Suzuki et al., 2012; Sierla et al., 2013; He et al., 2017; Shi et al., 2020; Zheng et al., 2021). The responses of ROS to various environmental stimuli may be attributed to different regulatory mechanisms of ROS production *via* NADPH oxidases (Baxter et al., 2014). On the other hand, the alteration of ROS scavengers affects the activity of ROS producers (Suzuki et al., 2013). For instance, *TaSOD2* encoding a superoxide dismutase enhances salt tolerance and NOX activity, and the inhibition of NOX erases the role of *TaSOD2* (Wang et al., 2016a). However, whether the role of 2-Cys peroxiredoxins in salt stress tolerance is associated with the alteration of ROS scavenging and production systems is not clear.

We previously bred a salt-tolerant wheat cultivar SR3 *via* asymmetric somatic hybridization with common wheat cultivar JN177 as the parent (Xia et al., 2003). In comparison with JN177, SR3 has superior photosynthesis efficiency under salt stress (Peng et al., 2009). To gain insight into the biochemical basis for salt tolerance of SR3, we performed a proteomic analysis and found there had obvious difference between the chloroplast proteomes of SR3 and JN177, and identified a 2-Cys peroxiredoxin TaBAS1 with differential abundances between SR3 and JN177 (Xu et al., 2016). In this work, we found that *TaBAS1* that offered salt tolerance. The role of *TaBAS1* in salt tolerance was associated with ROS producer NOX and 2-Cys Prx’s reductant NTRC. *TaBAS1* overexpression enhanced grain yield under salt stress without imposing the trade-offs between salt and yield, showing *TaBAS1* is an excellent candidate gene for germplasm improvement.

## Results

### TaBAS1 encoded a 2-Cys peroxiredoxin

**Figure 1:**
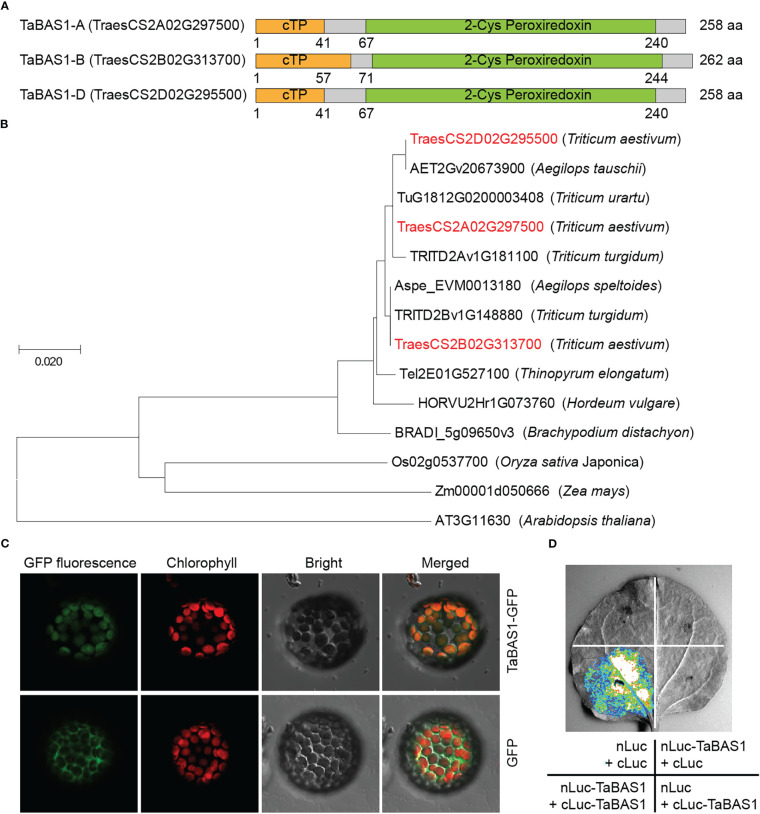
TaBAS1 is a 2-Cys peroxiredoxin. **(A)** The domain diagrams of TaBAS1 encoded by the alleles of **(A, B, D)** subgenomes. cTP: chloroplast targeting peptide. **(B)** The phylogenetic tree of TaBAS1 and the homologues of Triticeae species and Arabidopsis. **(C)** TaBAS1 localizes in the chloroplasts. **(D)** TaBAS1 interacts with itself.

In our previous comparative analysis on the chloroplast proteomes of wheat cultivars SR3 and JN177 (Xu et al., 2016), a protein with differential abundances between two cultivars was identified as typical 2-Cys peroxiredoxin (Prx) BAS1 (**Figure S1**). Using the peptide sequence of this protein as query to BLAST wheat genome, we isolated the coding sequence of BAS1 from wheat and named it *TaBAS1*. *TaBAS1* encoded a 2-Cys Prx, a subgroup of thioredoxin that belongs to thiol-specific antioxidant proteins. *TaBAS1* had three alleles locating in chromosome 2 of A, B and D subgenomes (**Figure 1A**). Three alleles shared high identity (98.1%) with each other, among which there had one amino acid polymorphism between TaBAS1-A and TaBAS1-D, and TaBAS1B had a 4-amino acid insertion and two amino acid polymorphisms in comparison with TaBAS1-A and TaBAS1-D. TaBAS1 possessed a chloroplast target sequence (cTP) in the N-terminus and a typical 2-Cys Prx domain in the C-terminus (**Figure 1A**). TaBAS1 and its homologues from Triticeae species and Arabidopsis were used for phylogenic analysis based on the peptide sequences. The result indicates that three TaBAS1 alleles were clustered closely with the homologues of their tetraploid and diploid progenitors (**Figure 1B**). They were grouped in a clade containing the homologues of Triticeae species including *Thinopyrum elongatum*, *Hordeum vulgare* and *Brachypodium distachyon*. Given that typical 2-Cys Prxs have been found to localize in the chloroplasts and TaBAS1 possessed a cTP domain, TaBAS1 fused with GFP as well as GFP alone was transiently expressed in the protoplasts to analyze it subcellular localization. The fluorescence of GFP dispersed in the cell and did not overlap with the red fluorescence of chlorophyll, whilst the fluorescence of TaBAS1 fused with GFP overlapped with the red fluorescence of chlorophyll (**Figure 1C**), indicating that TaBAS1 functions in the chloroplasts. Moreover, the split-luciferase complementation imaging (SLCI) assay indicated that TaBAS1 interacted with itself (**Figure 1D**), coinciding with the findings that 2-Cys peroxiredoxin can form homodimers.

### TaBAS1 was induced by salt and oxidative stress

**Figure 2:**
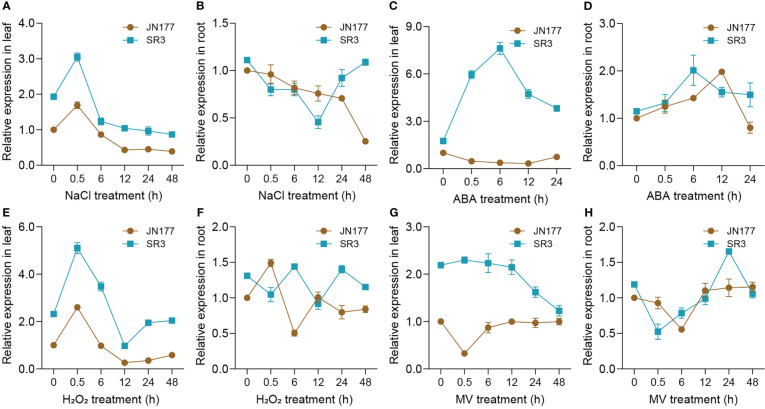
TaBAS1 is responsive to salt and oxidative stresses. **(A, B)** The response to NaCl in leaf and root; **(C, D)** The response to ABA in leaf and root; **(E, F)** The response to H2O2 in leaf and root; **(G, H)** The response to MV in leaf and root. Data are shown as mean and deviation (n = 4).

To primarily know whether *TaBAS1* participates in the response to salt stress, we measured its transcriptional profiles in the seedlings of SR3 and JN177 upon to NaCl treatment. In leaves, SR3 had higher transcription levels than JN177; *TaBAS1* was transiently induced at 0.5 h, but then decreased to the levels lower than the control condition after 6 h, and SR3 had higher transcript levels than JN177 in the whole treatment course (**Figure 2A**). In roots, the expression was declined gradually, but was resumed in SR3 after 12 h treatment (**Figure 2B**). The treatment of ABA, a stress associated phytohormone, drastically induced the expression of *TaBAS1* with the peak at 6 h in SR3 leaves, but had no obvious effect in JN177 leaves; *TaBAS1* was induced by ABA in the early period in roots, and the induction was quicker in SR3 than in JN177 (**Figures 2C, D**). The exposure to H_2_O_2_, a kind of ROS largely produced under stress, resulted in a transcription profile similar to that responsive to NaCl treatment in leaves, and the response appeared to be fluctuated in roots (**Figures 2E, F**). The herbicide methyl viologen (MV) induces ROS production in plants. In leaves, the abundance of *TaBAS1* transcripts kept constant but decreased after 12 h of MV treatment in SR3, while it was reduced after treatment for 0.5 h and then was resumed in JN177; in roots, the expression was decreased early but elevated in the following course of treatment in two cultivars (**Figures 2G, H**). These data indicate that *TaBAS1* is responsive to salt and oxidative stress.

### TaBAS1 enhanced salt tolerance

**Figure 3:**
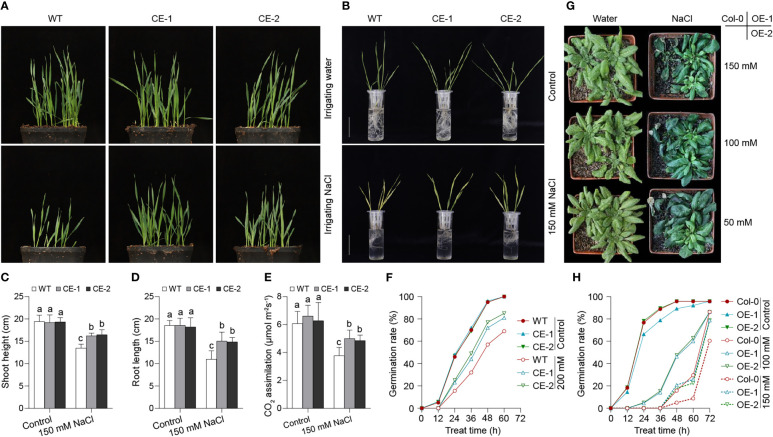
*TaBAS1* enhances salt tolerance in both wheat and Arabidopsis. **(A)**: Wheat seedlings grown in soil that were irrigated with and without NaCl solution. **(B)**: The comparison of wheat seedlings grown in liquid medium containing with and without NaCl. Bar = 5 cm. **(C, D)**: The plant height **(C)** and root length **(D)** of seedlings shown in panel **(B, E)**: The CO_2_ assimilation rate of seedlings grown in soil. **(F)**: The germination rate of wheat under none and NaCl treatment. **(G)**: The Arabidopsis seedlings grown in soil that were irrigated with different concentrations of NaCl solutions. **(H)**: The germination rate of Arabidopsis under none and NaCl treatment. Data are shown as mean and deviation [n = 10 in panels **(C–E)**; n = 3 in panels **(F)** and **(H)**]; in panels **(C–E)**, the difference among the samples under either control or stressful conditions is calculated with a one-way ANOVA analysis – Tukey comparison and the columns labeled without the same alphabet are significantly different (*P* < 0.05).

To evaluate the role of *TaBAS1* in salt tolerance, *TaBAS1* was overexpressed under the drive of ubiquitin promoter (*pUbi*) in wheat, and two independent transgenic lines producing more *TaBAS1* transcripts (CE) were selected for further analysis (**Figure S2A**). The CE lines and wildtype (WT) had similar growth capacity and comparable plant size when grown in the soil irrigated with water (**Figure 3A**). When irrigated with NaCl solution, the growth of WT seedlings was seriously restricted, but the growth restriction was alleviated in the CE lines, and the plant size of the CE lines was larger than that of WT. We further compared the salt tolerance of wheat seedlings grown in the liquid medium (**Figures 3B–D**). In the absence of NaCl treatment, the seedlings of the CE lines and WT were comparable, and they had similar shoot and root length. The addition of NaCl in the medium restricted the growth of both CE lines and WT, but the overexpression of *TaBAS1* obviously attenuated the restriction so that the CE lines had longer shoots and roots than WT. Because 2-Cys Prxs reduce H_2_O_2_ in the chloroplasts, we measured the CO_2_ assimilation rates of the seedlings in soil (**Figure 3E**). The CO_2_ assimilation rates are similar among WT and the CE lines when irrigated with water, but they were higher in the CE lines than in WT when supplied with NaCl solution. Consistently, the CE lines and WT had similar germination rates under the normal condition; when exposure to NaCl treatment, the germination was delayed, but the CE lines had faster germination rates than WT (**Figure 3F**).

**Figure 4:**
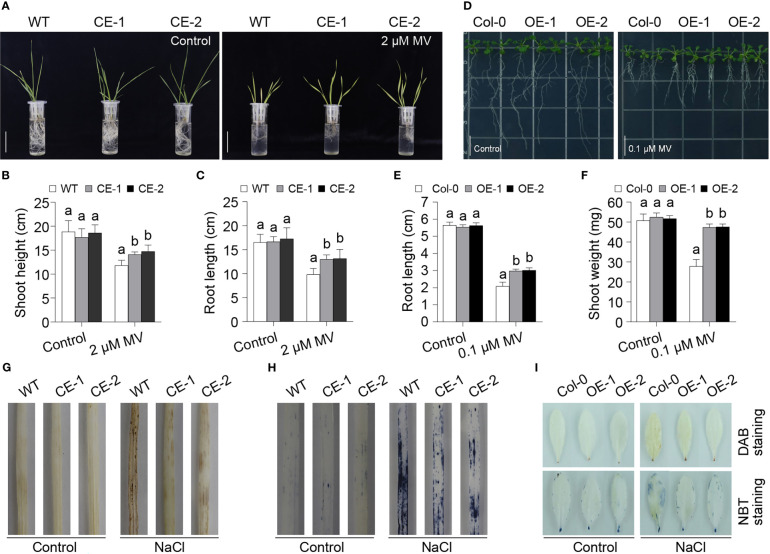
*TaBAS1* enhances tolerance to methyl viologen (MV) treatment. **(A)**: The growth capacity of wheat seedlings grown in the liquid medium containing with and without MV. Bar = 5 cm. **(B, C)**: The plant height **(B)** and root length **(C)** of seedlings shown in panel **(A, D)**: The phenotype of Arabidopsis seedlings grown in the agar plates containing with and without MV. Bar = 1 cm. **(E, F)**: The root length **(E)** and shoot weight **(F)** of seedlings shown in panel **(D, G, H)**: The DAB **(G)** and NBT **(H)** staining of wheat leaves. **(I)**: The DAB and NBT staining of Arabidopsis leaves. In panels **(B, C, E, F)**, data are shown as mean and deviation (n = 10); the difference among the samples under either control or stressful conditions is calculated with a one-way ANOVA analysis – Tukey comparison and the columns labeled without the same alphabet are significantly different (*P* < 0.05).

To further confirm the role of *TaBAS1* in salt tolerance, we ectopically overexpressed *TaBAS1* driven by CMV 35S promoter in Arabidopsis Col-0 to construct the overexpression (OE) lines (**Figure S2B**). In the soil irrigated with water, Col-0 and the OE lines had similar sizes of seedlings (**Figure 4G**). When the soil was irrigated with different concentrations of NaCl solutions, the growth of Col-0 seedlings was markedly restricted, but the OE lines exhibited quite vigorous growth ability. Consistently, as shown in our previous finding (Xu et al., 2016), the *TaBAS1* overexpression (OE) lines had comparable growth capacities to Col-0 in the solid agar medium, whilst in the presence of NaCl treatment, the growth was restricted in both Col-0 and the OE lines, but the restriction was obviously attenuated in the OE lines (**Figure S3**). Moreover, the CE lines and WT had similar germination rates under the unstressful condition, while in the presence of NaCl treatments, the CE lines had faster germination rates than WT (**Figure 3H**). These results indicate that *TaBAS1* enhances salt tolerance of both wheat and Arabidopsis.

### TaBAS1 enhanced the tolerance to oxidative stress

Given that 2-Cys Prxs are involved in ROS scavenging, so we further analyzed the role of *TaBAS1* in the tolerance to oxidative stress. As observed above, the CE lines had no difference from WT under the control condition (**Figures 4A–C**, **S4A–C**). The application of H_2_O_2_ restricted the growth of wheat seedlings, while the CE lines exhibited superior tolerance to H_2_O_2_ treatment, and they had longer shoots and roots than WT (**Figures S4A–C**). However, in Arabidopsis, the OE lines showed comparable tolerance to H_2_O_2_ treatments when compared with Col-0 (**Figures S4D–F**). Thus, we further analyzed the role of *TaBAS1 via* applying methyl viologen (MV) that can trigger ROS production. The exposure to MV restricted the growth of wheat seedings, but the CE lines had higher shoots and longer roots than WT (**Figures 4A–C**), showing that the CE lines had stronger tolerance to MV-induced oxidative stress. The application of MV to Arabidopsis seedlings phenocopied the effect on wheat seedlings, where the OE lines had superior growth ability, larger fresh weights and longer roots in comparison with Col-0 (**Figures 4D–F**). To further confirm the association between the role of *TaBAS1* in salt tolerance and ROS scavenging, we measured the ROS levels in the leaves. The 3’-diaminobenzidine (DAB) staining assay representing H_2_O_2_ level showed that the CE lines and WT had similar H_2_O_2_ levels under the control condition (**Figure 4G**). NaCl treatment elevated the H_2_O_2_ levels in all samples, with higher contents in WT than in the CE lines. The Nitrotetrazolium Blue chloride (NBT) staining assay indicating O_2_^-^ content got the similar result that the CE lines had comparable O_2_^-^ levels under the control condition but lower levels upon salt stress in comparison with WT (**Figure 4H**). The parallel analysis in Arabidopsis leaves further indicated that *TaBAS1* ectopic expression reduced ROS levels under salt stress (**Figure 4I**).

### The role of TaBAS1 in salt tolerance was associated with ROS production

**Figure 5:**
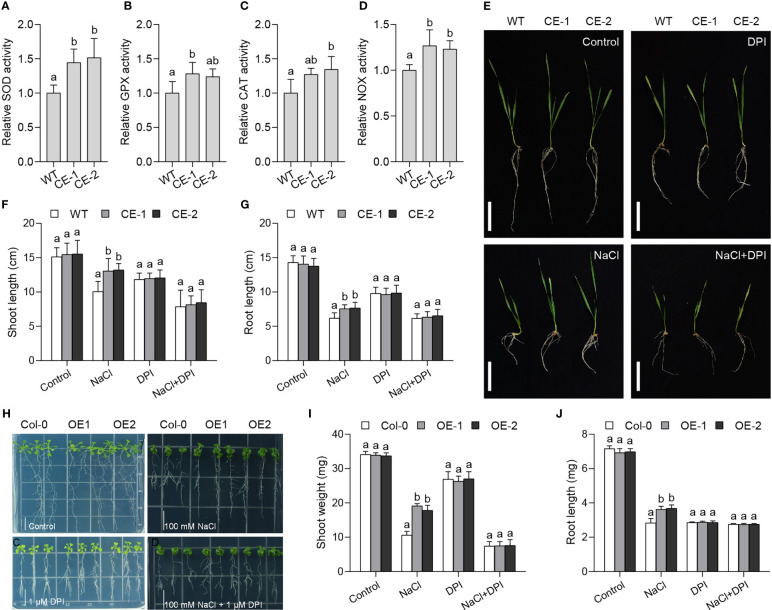
The role of *TaBAS1* in salt tolerance is associated with NADPH oxidase. **(A–D)**: The relative activities of superoxide dismutase **(A)**, glutathione peroxidase **(B)**, catalase **(C)** and NADPH oxidase **(D)** in the leaves of wheat seedlings. **(E)**: The addition of NADPH oxidase inhibitor DPI erases the role of *TaBAS1* in salt tolerance in wheat. Bar = 5 cm. **(F, G)**: The shoot length **(F)** and root length **(G)** of seedlings shown in panel **(E, H)**: The presence of DPI abolishes the role of *TaBAS1* in salt tolerance in Arabidopsis. Bar = 1 cm. **(I, J)**: The shoot weight **(I)** and root length **(J)** of seedlings shown in panel **(H)** Data are shown as mean and deviation (n = 5 in panels A-D; n = 10 in panels **(F, G, I, J)**); the difference among the samples under either control or stressful conditions is calculated with a one-way ANOVA analysis – Tukey comparison and the columns labeled without the same alphabet are significantly different (*P* < 0.05).

To know whether TaBAS1 affects the ROS homeostasis system, we measured the activities of some ROS scavenging enzymes. The activities of superoxide dismutase (SOD), glutathione peroxidase (GPX) and catalase (CAT) of the CE lines were higher than those of WT (**Figures 5A–C**), showing *TaBAS1* overexpression can promote the ROS scavenging system to enhance ROS removal ability. The change of ROS scavengers often influences ROS producers such as NOX (Wang et al., 2016a). We found that the CE lines had pronounced NOX activities than WT (**Figure 5D**), and *TaBAS1* overexpression improved the transcription of NOX genes in wheat and Arabidopsis (**Figure S5**), indicating that *TaBAS1* overexpression elevates not only ROS scavenging but also production. To further analyze the association between *TaBAS1* and ROS homeostasis, we evaluated the effect of NOX inhibitor diphenyleneiodonium (DPI) on the role of *TaBAS1* in salt tolerance. The application of DPI alone restricted the growth of the CE lines and WT with similar restriction extent (**Figures 5E–G**). As found above, the CE lines had superior growth status than WT under NaCl treatment. When exposed to NaCl and DPI together, the growth of both the CE lines and WT was obviously restricted, and there had no difference between WT and the CE lines. The salt tolerance by *TaBAS1* ectopic expression in Arabidopsis was also lost in the presence of DPI (**Figures 5H–J**). The parallel analysis showed that the presence of DPI to inhibit NOX activity also abolished the role of *TaBAS1* in the tolerance to MV in both wheat and Arabidopsis (**Figure S6**). These results indicate that the role of *TaBAS1* in drought tolerance is associated with NOX.

### The involvement of NTRC in salt tolerance of TaBAS1

**Figure 6:**
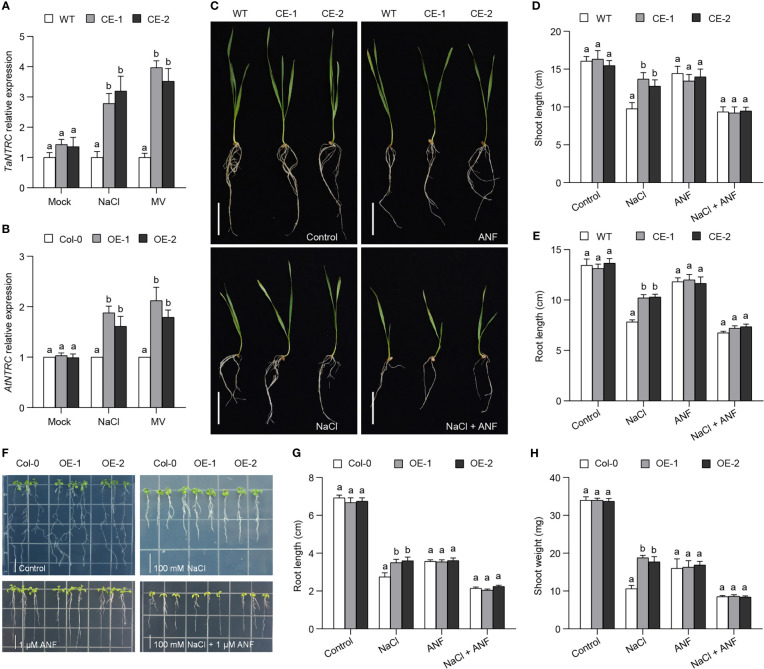
The enhancement of salt tolerance by *TaBAS1* is dependent on NADPH-dependent thioredoxin reductase C (NTRC). **(A, B)**: The relative expression of NTRC genes in wheat **(A)** and Arabidopsis **(B)**. **(C)**: The inhibition of NTRC activity by adding NTRC inhibitor ANF restricts the role of *TaBAS1* in salt tolerance in wheat. Bar = 5 cm. **(D, E)**: The shoot length **(D)** and root length **(E)** of seedlings shown in panel **(C, F)**: The inhibition of NTRC activity erases the role of *TaBAS1* in salt tolerance in Arabidopsis. Bar = 1 cm. **(G, H)**: The root length **(G)** and shoot weight **(H)** of seedlings shown in panel **(F)** Data are shown as mean and deviation (n = 4 in panels **(A, B)**; n = 10 in panels **(D, E, G, H)**; the difference among the samples under either control or stressful conditions is calculated with a one-way ANOVA analysis – Tukey comparison and the columns labeled without the same alphabet are significantly different (*P* < 0.05).

It has proved that NADPH-thioredoxin reductase C (NTRC) is involved in the machinery of 2-Cys Prxs through controlling the redox balance of 2-Cys Prxs (Muthuramalingam et al., 2009). In comparison with WT, the CE lines produced more abundance of *NTRC* transcripts (**Figure 6A**). Similarly, the expression level of *AtNTRC* was also higher in the OE lines than in Col-0 (**Figure 6B**). These data showed that *TaBAS1* overexpression improved the expression of *NTRC* genes. We then analyzed the association of NTRC with the role of *TaBAS1* in salt tolerance using NTRC inhibitor auranofin (ANF). ANF application inhibited the growth of the CE lines and WT with the same extent; NaCl treatment restricted the growth of all samples, with less restriction strength in the CE lines; when treated with NaCl and ANF together, the CE lines had comparable growth status, shoot and root length to WT (**Figures 6C–E**). The analysis of Arabidopsis seedlings also found that the inhibition of NTRC activity by ANF application restricted the growth of the OE lines and Col-0, and caused more severe growth restriction in the presence of NaCl with no difference between the OE lines and Col-0 (**Figures 6F–H**). Furthermore, the stronger growth ability of wheat and Arabidopsis seedlings by *TaBAS1* overexpression under MV treatment was also removed when ANF was applied (**Figure S7**). These results indicate that NTRC is involved in the machinery of TaBAS1 during the response to salt stress.

### TaBAS1 enhanced grain yield under salt stress

**Figure 7:**
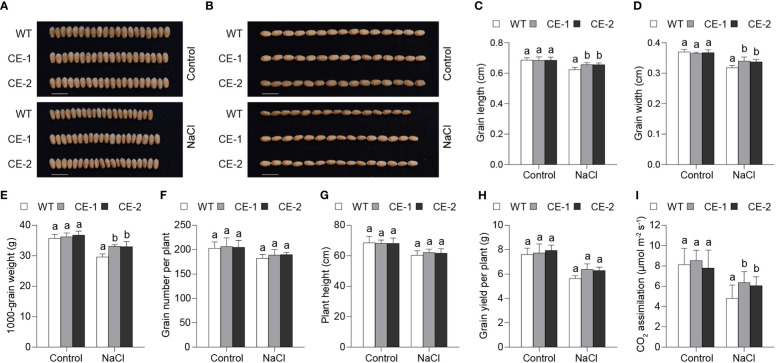
*TaBAS1* enhances grain yield of wheat under salt stress. **(A, B)**: The comparison of wheat grain sizes under the control and salt-stressed conditions. Bar = 1 cm. **(C, D)**: The grain length **(C)** and width **(D)** shown in panels **(A, B, E)**: *TaBAS1* increases 1000-grain weight under salt stress. **(F)**: The comparison of grain numbers per plant. **(G)**: The comparison of plant height. **(H)**: *TaBAS1* enhances grain yields under salt stress. **(I)**: *TaBAS1* improves CO_2_ assimilation rate under drought stress. Data are shown as mean and deviation [n = 6 in panels **(C-F)**; n = 10 in panels **(G–I)**]; the difference among the samples under either control or stressful conditions is calculated with a one-way ANOVA analysis – Tukey comparison and the columns labeled without the same alphabet are significantly different (*P* < 0.05).

Given that *TaBAS1* enhanced salt tolerance of wheat seedlings, we further analyzed the role of *TaBAS1* overexpression in grain yield of wheat planted in the pots. The CE lines and WT had similar grain sizes (width and length) when the plants were irrigated with water (**Figures 7A–D**). When the plants were irrigated with NaCl solution at the jointing stage, the grain sizes were reduced, but the CE lines had large the grain sizes than WT. Consistently, 1000-grain weights (TGWs) were comparable among the CE lines and WT under the water-irrigated condition, but TGWs of the CE lines were larger by nearly 12% than that of WT under the salt-stressful condition (**Figure 7E**). Under either water-irrigated or salt-stressful condition, the spike numbers per plant and plant heights of the CE lines were not different from those of WT (**Figures 7F, G**). Consequently, in comparison with WT, the CE lines had similar grain yields under the water-irrigated condition, but had higher yields by 11.7% - 13.5% under salt stress (**Figure 7H**). Under the control condition, there had no difference in CO_2_ assimilation rates between the CE lines and WT; the irrigation of NaCl solution reduced CO_2_ assimilation rates of all plants, among which the CE lines had higher CO_2_ assimilation rates than WT (**Figure 7I**). These results indicate that *TaBAS1* overexpression enhances grain yield under salt stress, but has no effect on the plant growth and grain yield under the control condition. Thus, *TaBAS1* does not impose the trade-offs between yield and tolerance.

## Discussion

### TaBAS1 is a salt tolerant gene with potential in molecular breeding

Salt and other abiotic stresses induce comprehensive physiological alteration, which in turn enhances the adaptation capacity of plants to the stresses. Thus, the genetic manipulation of genes directly modulating the physiological response would efficiently enhances the tolerance to salt. In this work, we identify a chloroplast localized 2-Cys Prx encoding gene *TaBAS1* (**Figure 1**), and its overexpression obviously enhances salt tolerance in wheat and Arabidopsis (**Figures 3**, **S3**) (Xu et al., 2016). In line with the reports that the enhancement of salt tolerance by 2-Cys Prxs from *Suaeda salsa* L. and *Tamarix hispida* (Jing et al., 2006; Wang et al., 2020), our data indicate that the role of 2-Cys-Prxs in salt tolerance is conserved in different plants. Moreover, *TaBAS1* enhances grain yield of wheat under salt stress, but has no adverse effect on the growth and yield under the control condition (**Figure 7**), exhibiting its potential in the germplasm improvement of wheat without imposing the trade-offs between yield and tolerance. Moreover, 2-Cys Prxs have been proved to enhance the tolerance to cold, heat and drought (Bhatt and Tripathi, 2011; Vogelsang and Dietz, 2022). Thus, the genes of 2-Cys Prxs and/or other components that directly modulating the level of H_2_O_2_ (ROS) and other basic physiological bases of the adaption to abiotic stress could be used for improving broad-spectrum tolerance to abiotic stresses.

### The role of TaBAS1 is associated with ROS homeostasis

2-Cys Prxs possess peroxidase activity on a variety of peroxide substrates, and contribute to the tolerance to oxidative stress (König et al., 2002; Vidigal et al., 2015; Li et al., 2016; Mishra et al., 2021). Consistently, *TaBAS1* enhances the tolerance to H_2_O_2_ and MV-induced oxidative stress, reduces ROS levels, and increases CO_2_ assimilation rate under salt stress (**Figures 3E**, **4**, **S4**, **7I**), further confirming the physiological basis of 2-Cys Prxs in the response to salt (abiotic) stress. Note that *TaBAS1* overexpression enhances the tolerance to H_2_O_2_ in wheat but not Arabidopsis (**Figure S4**), which may owe to the difference in the response threshold to H_2_O_2_ treatment that need to be studied in the future. Moreover, *TaBAS1* promotes the activities of ROS scavenging enzymes SOD, GPX and CAT (**Figures 5A–C**), consistent with the previous studies that 2-Cys Prxs elevate these ROS scavenging enzymes in other plants (Pulido et al., 2010; Wang et al., 2020; Xu et al., 2022). This indicates that 2-Cys Prxs can affect the ROS scavenging system to efficiently remove excessive ROS together. It has been suggested that the peroxidase activity of 2-Cys Prxs can be compensated by the other antioxidant systems of the chloroplasts, such as the ascorbate-glutathione cycle in combination with superoxide dismutase (Pulido et al., 2010). In line with that wheat SOD gene *TaSOD2* promotes the activities of GPX, APX and SOD (Wang et al., 2016a), our results show the close association among the components of ROS scavenging machinery in plants. On the other hand, NOX is the crucial component of ROS production system. Here, we find *TaBAS1* elevates the activities of NOX, and accelerates the expression levels of NOX encoding genes (**Figures 5D**, **S5**). Moreover, our previous study showed that SR3 has higher ROS level and NOX activity compared with JN177 under both control and salt stress conditions (Liu et al., 2004; Peng et al., 2009). In combination with these data, *TaBAS1* offers salt tolerance through ROS homeostasis modulation, which is achieved *via* pushing both ROS scavenging and production systems. As toxic and signaling molecules, a suitable level of ROS modulated by the balance between ROS production and scavenging systems is required (Mittler et al., 2004). NOX plays crucial roles in abiotic stress tolerance and antioxidant defense capacity (Torres and Dangl, 2005; Suzuki et al., 2012; Sierla et al., 2013; He et al., 2017; Shi et al., 2020). For example, the mutation of rice NOX genes *OsRbohA* and *OsRbohB* reduced drought tolerance, but its overexpression enhanced ROS level and drought tolerance (Wang et al., 2016b; Shi et al., 2020); the inhibition of NOX activity by the inhibitor DPI significant reduces salt tolerance of wheat (Zheng et al., 2021). The contribution of wheat SOD gene *TaSOD2* in salt tolerance is associated with NOX, and the inhibition of NOX activity by adding DPI or the mutation of NOX genes removes the role of *TaSOD2* (Wang et al., 2016a). Here, the inhibition of NOX activity abolished the role of *TaBAS1* in salt and oxidative tolerance (**Figures 5**, **S6**). In line with the increase of the activities of SOD, GPX and CAT in the *TaBAS1* overexpression lines, 2-Cys Prxs seem to enhance salt tolerance *via* modulating not only ROS scavengers but also ROS producers to alter the ROS homeostasis.

### The contribution of TaBAS1 needs the resumption ability of its reduction status

The reduction of peroxidatic Cys is necessary for the activity of 2-Cys Prxs, and NTRC plays important roles in the redox balance of the 2-Cys Prxs because it is an efficient reductant of 2-Cys Prxs (Moon et al., 2006; Pérez-Ruiz et al., 2006; Alkhalfioui et al., 2007; Ojeda et al., 2018). NTRC redox system is integrated by the redox balance of the 2-Cys Prxs, which controls the redox regulatory network of the chloroplasts (Pérez-Ruiz et al., 2017). The mutation of *NTRC* decreases the levels of 2-Cys Prxs (Ojeda et al., 2018). Here, we find that the inhibition of NTRC activity erases the role of *TaBAS1* in the tolerance to salt and oxidative stress (**Figures 6**, **S7**), firstly confirming that the enhancement of salt tolerance by 2-Cys Prxs is dependent on NTRC. Moreover, *TaBAS1* promotes the expression of NTRC genes (**Figures 6A, B**). This may result in NTRC accumulation to ensure the reduction capacity for 2-Cys Prxs, which promotes their activities to enhance salt tolerance. It is noted that the increase in the expression of NTRC genes by *TaBAS1* overexpression was found in the present of salt and oxidative stress (**Figures 6A, B**). Because the ROS level is elevated under salt stress, the orchestration between NTRC and 2-Cys Prxs appears to be dependent on ROS status. Under the stressful condition, ROS are over-accumulated, which causes the oxidation of 2-Cys Prxs and therefore needs more NTRC to reduce.

In summary, *TaBAS1* exhibits the potential in wheat molecular breeding because of not imposing the trade-offs between salt and yield. The performance of *TaBAS1* in salt tolerance is closely associated with intracellular ROS homeostasis and the cycle of 2-Cys Prx redox status, suggesting the complication of ROS modulatory scenario in plants.

## Materials and methods

### Cloning of TaBAS1 coding sequence

The peptide sequence of the differentially expressed protein spots identified by the mass spectrometry were subjected to BLAST the wheat genome to obtain the gene (*TaBAS1*) encoding the peptide. The coding sequence (CDS) of *TaBAS1* was cloned from the cDNA of wheat cultivar SR3 using the primers listed in **Supplemental Table S1**. The PCR procedure consisted of a 5 min denaturation at 95 °C, followed by 35 cycles of 94 °C/30 s, 58 °C/50 s and 72 °C/60 s, with a final extension of 72 °C/10 min. The homologues of *TaBAS1* from Triticeae species and Arabidopsis were extracted with the online Triticeae-Gene Tribe system, and the peptide sequences of these homologues were used for phylogenic analysis based on the neighbour joining method with the software packages CLUSTAL X and MEGA4.1 software (Kumar et al., 2004; Larkin et al., 2007).

### The construction of TaBAS1 overexpression lines of wheat and Arabidopsis

The vector ligating *TaBAS1* driven by the ubiquitin promoter was introduced into a common wheat cultivar YM158 using the *Agrobacterium tumefaciens* – mediated shoot apical meristem transformation method (Zhao et al., 2006). The vector ligating *TaBAS1* driven by the CaMV 35S promoter was transformed into Arabidopsis Col-0 using the floral dip method (Clough and Bent, 1998). The transcription of the transgene in the stably transgenic lines of wheat and Arabidopsis was detected by real-time PCR with total cDNA as template.

### The treatment of wheat and Arabidopsis seedlings

To detect the transcriptional profiles of *TaBAS1*, seedlings of SR3 and JN177 at three-leaf stage that were grown in half strength Hoagland’s liquid medium under a 16 h photoperiod at 22 °C were transferred into the liquid medium containing none, 200 mM NaCl, 10 mM H_2_O_2_, 100 μM ABA and 0.1 μM MV for up to 48 h. After different times within the treatment course, the leaves and roots of the seedlings were sampled for RNA extraction using the Trizol method. The RNA samples were reversely transcribed into cDNA for real-time quantitative PCR (qPCR). The description of these and the following treatments are listed in **Supplemental Table 2**.

Seven-day-old seedlings of wheat were transferred into half strength Hoagland’s liquid medium with none, 150 mM NaCl, 20 mM H_2_O_2_, 2 μM MV for seven days under the growth condition as above. Seven-day-old seedlings grown in the soil were irrigated with 200 mL water or 150 mM NaCl solution every two days for three times, and the seedlings were recorded after seven days. Wheat seedlings were placed in the liquid medium containing none, 2 μM diphenyliodonium (DPI, NOX inhibitor), 2 μM DPI with 150 mM NaCl or 2 μM MV for seven days to analyze the role of NOX on salt tolerance. Wheat seedlings were placed in the liquid medium containing none, 2 μM auranofin (ANF, NTRC inhibitor), 2 μM ANF with 150 mM NaCl or 2 μM MV for seven days to analyze the role of NTRC. The wheat seeds were half-soaked in water or 200 mM NaCl solution in the Petri dishes at room temperature, and the germinated seeds were counted ever 12 h for up to 72 h. The germination rate was defined as the number the germinated seeds divided by the number of total seeds.

Arabidopsis seeds were plated on half strength Murashige and Skoog (MS) agar medium, placed in the dark at 4 °C for 2 days to break dormancy, and transferred to a 16 h photoperiod at 22 °C for three days. The seedlings were then re-plated on half strength MS agar medium supplemented with 0, 50 or 100 mM NaCl, with 0, 0.5 or 1 mM H_2_O_2_ and with 0 or 0.1 μM MV for ten days. The seedlings were transferred into the MS agar medium containing 100 mM NaCl, 1 μM DPI, 1 μM DPI with 100 mM NaCl, 0.1 μM MV, 1 μM DPI with 0.1 μM MV for ten days to analyze the role of NOX. The seedlings were transferred into the MS agar medium containing 100 mM NaCl, 1 μM ANF, 1 μM ANF with 100 mM NaCl, 0.1 μM MV, 1 μM ANF with 0.1 μM MV for ten days to analyze the role of NTRC. The sterile Arabidopsis seeds were plated on the solid half strength MS agar plates containing 0, 100 or 150 mM NaCl, and placed in the dark at 4°C for 2 days to break dormancy; the seeds were then transferred to 22°C for germination, and the germinated seeds were counted ever 12 h for up to 72 h. The germination rate was defined as the number the germinated seeds divided by the number of total seeds.

### Real-time PCR analysis

Total RNA was extracted from the leaves of both wheat and Arabidopsis seedlings using the Trizol reagent (Invitrogen), and treated with DNAase I. The cDNA strand was synthesized using the M-MLV reverse transcription system kit (Invitrogen). The cDNA was used for real-time PCR in a 20 μL solution containing 10 μL SYBR Premix Ex Taq mix (Takara), 0.2 μM forward and reverse primers respectively, 1 μL the cDNA, and the cycling regime comprised 95 °C for 2 min, 45 cycles of 95 °C for 10 s, 60 °C for 20 s, 72 °C for 20 s. Relative gene expression levels were detected using the 2-DDCT method (Livak and Schmittgen 2001). Wheat TUBULIN gene (TraesCS3D02G326900) and the Arabidopsis TUBULIN gene (AT1G04820) were used as the internal references.

### Subcellular localization

The coding sequence of*TaBAS1* without the stop codon was ligated into the vector 326 to construct pBI221-TaBAS1 that can expression TaBAS1-GFP fused protein. Either pBI221-TaBAS1 or pBI221 was introduced into the wheat protoplasts by the PEG-mediated transfection (Shan et al., 2014). Then the protoplasts were incubated in dark at 25°C for 16–24 h. The confocal images were finally captured using the ZEISS LSCM 900 system.

### Luciferase imaging

The *TaBAS1* coding sequence was ligated into the vector JW771 to construct N-terminus luciferase (nLuc)-TaBAS1 fused ORF vector, and it was ligated into the vector JW772 to construct C-terminus luciferase (cLuc)-TaBAS1 ORF vector. Pairs of nLUC and cLUC constructs were transformed into *Ag. tumefaciens* strain EHA105 and the transformants were infiltrated into the leaves of *N. benthamiana* plants under the condition of 16-h photoperiod at 22°C for three days. The leaves were sampled and incubated with 1 mM D-luciferin-free acid (GoldBio) dissolved in 0.01% (v/v) Triton X-100 and incubated for 5 min in the dark. The fluorescence image was acquired with a CCD camera.

### DAB and NBT staining

Wheat seedlings at the three-leaf-stage were transferred into half strength Hoagland’s liquid medium with none and 200 mM NaCl for 2 days. 3-week-old Arabidopsis seedlings that planted in soils were irrigated with 100 mL water or 100 mL of 150 mM NaCl for one day. After treatment, the leaves of wheat and Arabidopsis were sampled for measuring H_2_O_2_ level by DAB (3’-diaminobenzidine) staining assay (Asselbergh et al., 2007) and measuring O_2_^-^ level by NBT (Nitrotetrazolium Blue chloride) staining assay. For DAB staining, the leaves were stained by floating in 1 mg/mL DAB-HCl (pH 4) at 28 °C in dark for 24 h for wheat leaves and 8 h for Arabidopsis leaves. For NBT staining, the leaves were floated in 10 mM KH_2_PO_3_/K_2_HPO_3_ (pH 7.6) containing 0.5 mg/mL NBT at 28 °C in dark for 24 h for wheat leaves and 3 h for Arabidopsis leaves. After staining, chlorophyll was removed with 95% ethanol in boiling bath.

### Physiological indices measurement

Wheat seedlings at three-leaf-stage and the 2-week-old Arabidopsis seedlings were sampled, and homogenized in the buffer solution containing 1 mL 50 mM KH_2_PO_4_, 0.1 mM EDTA, and 0.3% (w/v) Triton X-100 at 4 °C. After centrifugation, the supernatants were used for measuring the activities of SOD, GPX, CAT, and NOX. SOD activity was measured using the SOD detection kit according to the manual (Category number: S0109, Beyotime Institute of Biotechnology, China). CAT activity was determined by monitoring the decrease in absorbance at 240 nm of H_2_O_2_ for 1 min at 25 °C using the detected kit (Category number: S0051, Beyotime Institute of Biotechnology, China). GPX activity was assayed using by monitoring the decrease in absorbance at 340 nm of the reaction system using the detection kit (Category number: S0056, Beyotime Institute of Biotechnology, China). The NOX activity was measured according to the previous method (Grace and Logan, 1996).

### The measurement of yield

The seeds of the CE lines and WT were sown in plastic pots containing equal weights of soils in the green house. One seedling was retained in one pot, and the other seedlings were removed. At the beginning of jointing, 200 mL of 150 mM NaCl solution were irrigated into the pot every two days for four times. After harvesting, the grain yield and the yield associated indices including grain size and width, 1000-grain weight, grain number per spike were measured. At grain filling period, CO_2_ assimilation rate was measured with an LI-6400XT Portable Photosynthesis System (Li-Cor, USA).

### Statistical analysis

The normal distribution of the data was analyzed with the Shapiro-Wilk test. The difference of the indices among three samples was calculated with one-way ANOVA test, and the *post-hoc* comparison was conducted with the Tukey method at the significance level of 0.05.

## Data availability statement

The original contributions presented in the study are included in the article/**Supplementary Material**. Further inquiries can be directed to the corresponding author.

## Author contributions

MW and GMX conceived the work. GLX, MZ, JZ, QL, QW, ZC and MW conducted the experiment and analyzed the data. MW and GLX wrote the paper. All authors read the manuscript. All authors contributed to the article and approved the submitted version.

## Supplementary material

Supplementary Figure 1 The identification of TaBAS1. **(A)**: The protein spots with differential abundances between SR3 and JN177 in the two-dimensional electrophoresis map. **(B)**: The identification of TaBAS1 using mass spectrometry data in Mascot (https://www.matrixscience.com/cgi/search_form.pl?FORMVER=2&SEARCH=PMF). **(C)**: The matched peptides using mass spectrometry data in Mascot.

Supplementary Figure 2 The expression levels of *TaBAS1* in transgenic wheat **(A)** and Arabidopsis **(B)**. Data are shown as mean and deviation (n = 4); the difference among the samples is calculated with a one-way ANOVA analysis – Tukey comparison and the columns labeled without the same alphabet are significantly different (*P* < 0.05).

Supplementary Figure 3
*TaBAS1* enhanced salt tolerance of Arabidopsis. **(A, B)**: The phenotypes of Arabidopsis seedlings under the control and salt-stressed conditions. Bar = 1 cm. **(C, D)**: The shoot weight **(C)** and root length **(D)** of seedlings shown in panels **(A, B)**. Data are shown as mean and deviation (n = 10); in panels **(C, D)**, the difference among the samples under either control or stressful conditions is calculated with a one-way ANOVA analysis – Tukey comparison and the columns labeled without the same alphabet are significantly different (*P* < 0.05).

Supplementary Figure 4
*TaBAS1* enhances tolerance to H_2_O_2_ treatment in wheat. **(A)**: Wheat seedlings grown in the liquid medium containing with and without H_2_O_2_. **(B, C)**: The plant height **(C)** and root length **(D)** of seedlings shown in panel **(A, D)**: The phenotype of Arabidopsis seedlings grown in the agar plates containing with and without H_2_O_2_. Bar = 1 cm. **(E, F)**: The shoot weight **(E)** and root length **(F)** of seedlings shown in panel **(D)**. Data are shown as mean and deviation (n = 10); in panels **(B, C, E, F)**, the difference among the samples under either control or stressful conditions is calculated with a one-way ANOVA analysis – Tukey comparison and the columns labeled without the same alphabet are significantly different (*P* < 0.05).

Supplementary Figure 5 The expression of NADPH oxidase encoding genes in wheat and Arabidopsis. **(A, B)**: The expression in wheat. **(C, D)**: The expression in Arabidopsis. Data are shown as mean and deviation (n = 4); the difference among the samples is calculated with a one-way ANOVA analysis – Tukey comparison and the columns labeled without the same alphabet are significantly different (*P* < 0.05).

Supplementary Figure 6 The role of *TaBAS1* in oxidative tolerance is associated with NADPH oxidase. **(A)**: The addition DPI erases the role of *TaBAS1* in MV tolerance of wheat. Bar = 5 cm. **(B, C)**: The shoot length **(B)** and root length **(C)** of seedlings shown in panel **(A, D)**: The presence of DPI abolishes the role of *TaBAS1* in MV tolerance of Arabidopsis. Bar = 1 cm. **(E, F)**: The shoot weight **(E)** and root length **(F)** of seedlings shown in panel **(D)**. Data are shown as mean and deviation (n = 10); in panels **(B, C, E, F)**, the difference among the samples under either control or stressful conditions is calculated with a one-way ANOVA analysis – Tukey comparison and the columns labeled without the same alphabet are significantly different (*P* < 0.05).

Supplementary Figure 7 The enhancement of MV tolerance by *TaBAS1* is dependent on NTRC. **(A)**: The addition of ANF removes the role of *TaBAS1* in MV tolerance of wheat. Bar = 5 cm. **(B, C)**: The shoot length **(D)** and root length **(E)** of seedlings shown in panel **(A, D)**: The inhibition of NTRC activity erases the role of *TaBAS1* in MV tolerance of Arabidopsis. Bar = 1 cm. **(E, F)**: The shoot weight **(E)** and root length **(F)** of seedlings shown in panel **(D)**. Data are shown as mean and deviation (n = 10); in in panels **(B, C, E, F)**, the difference among the samples under either control or stressful conditions is calculated with a one-way ANOVA analysis – Tukey comparison and the columns labeled without the same alphabet are significantly different (*P* < 0.05).
