# Supplementary material for: TaBAS1 encoding a typical 2-Cys peroxiredoxin enhances salt tolerance in wheat
Source: Front Plant Sci. 2023 Mar 14;14:1152375. doi: 10.3389/fpls.2023.1152375 (PMC10043318; doi:10.3389/fpls.2023.1152375)
Supplement: Supplementary Figure 1 — The identification of TaBAS1. (A): The protein spots with differential abundances between SR3 and JN177 in the two-dimensional electrophoresis map. (B): The identification of TaBAS1 using mass spectrometry data in Mascot (https://www.matrixscience.com/cgi/search_form.pl?FORMVER=2&SEARCH=PMF). (C): The matched peptides using mass spectrometry data in Mascot. [file DataSheet_1.docx]

**
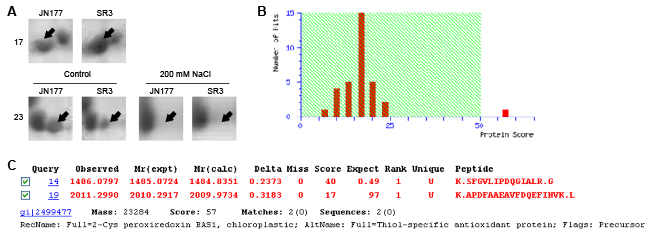
**

**Supplementary Figure 1**

**
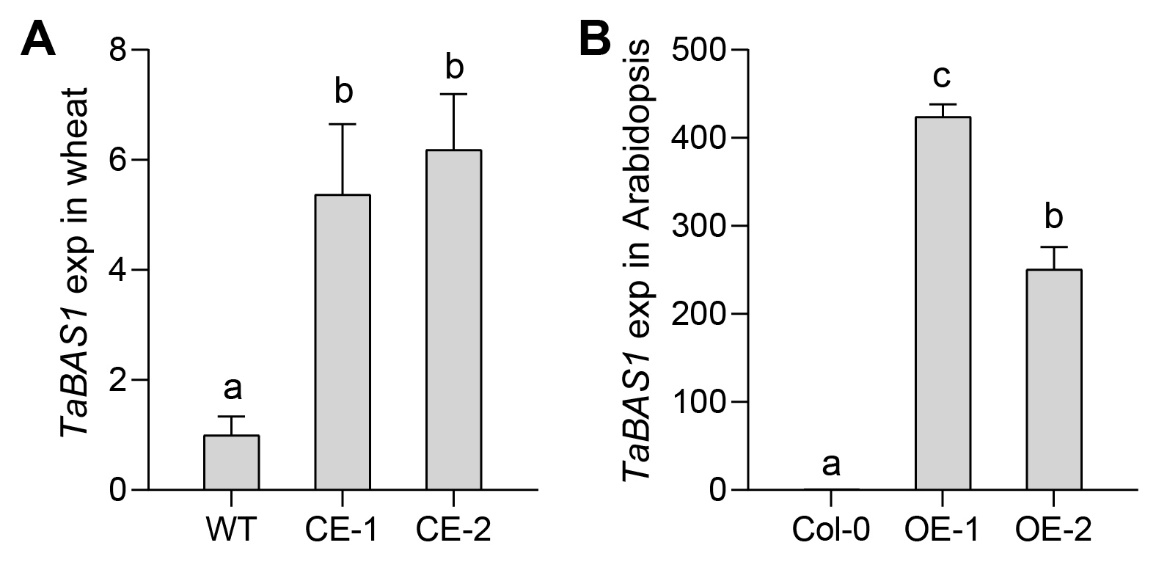
**

**Supplementary Figure 2**

**
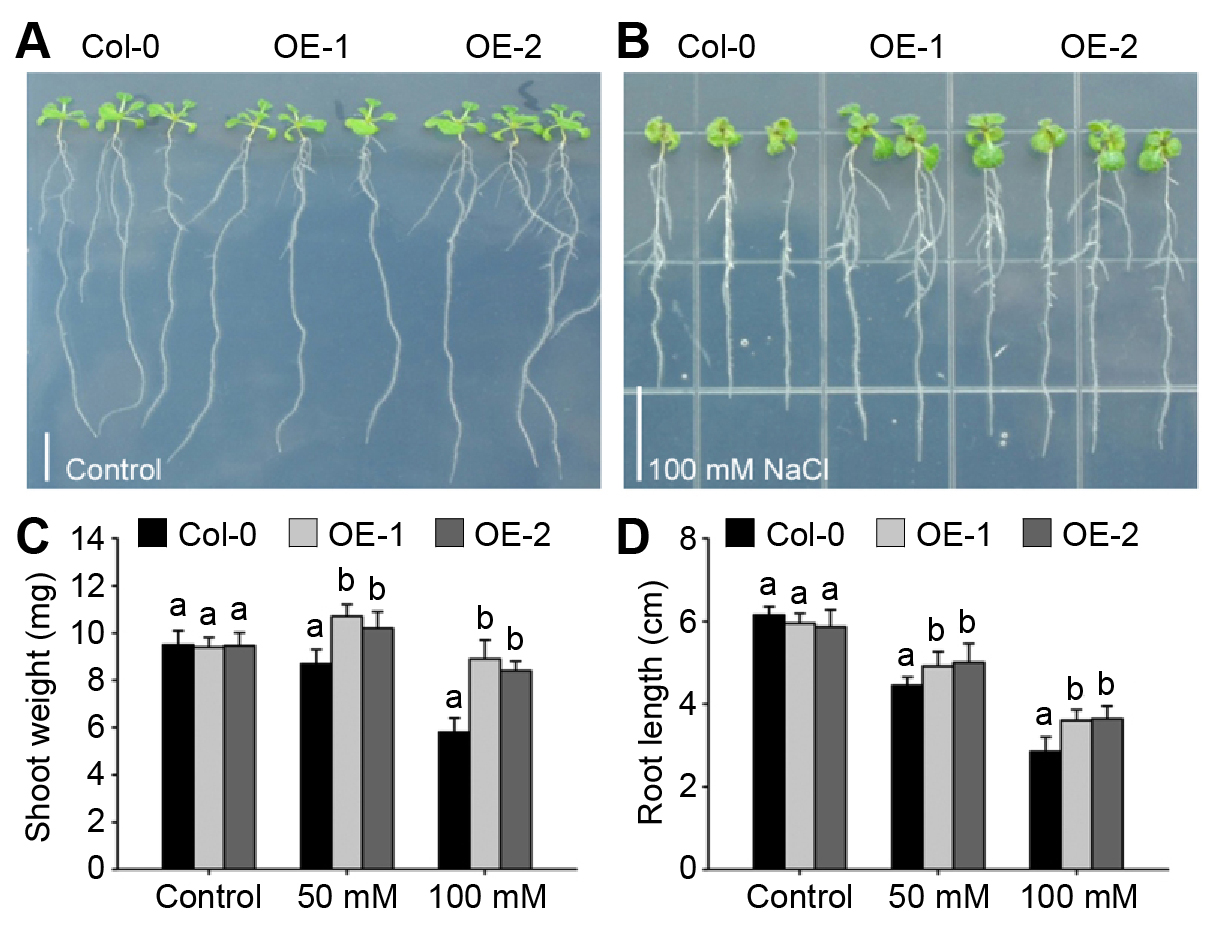
**

**Supplementary Figure 3**

**
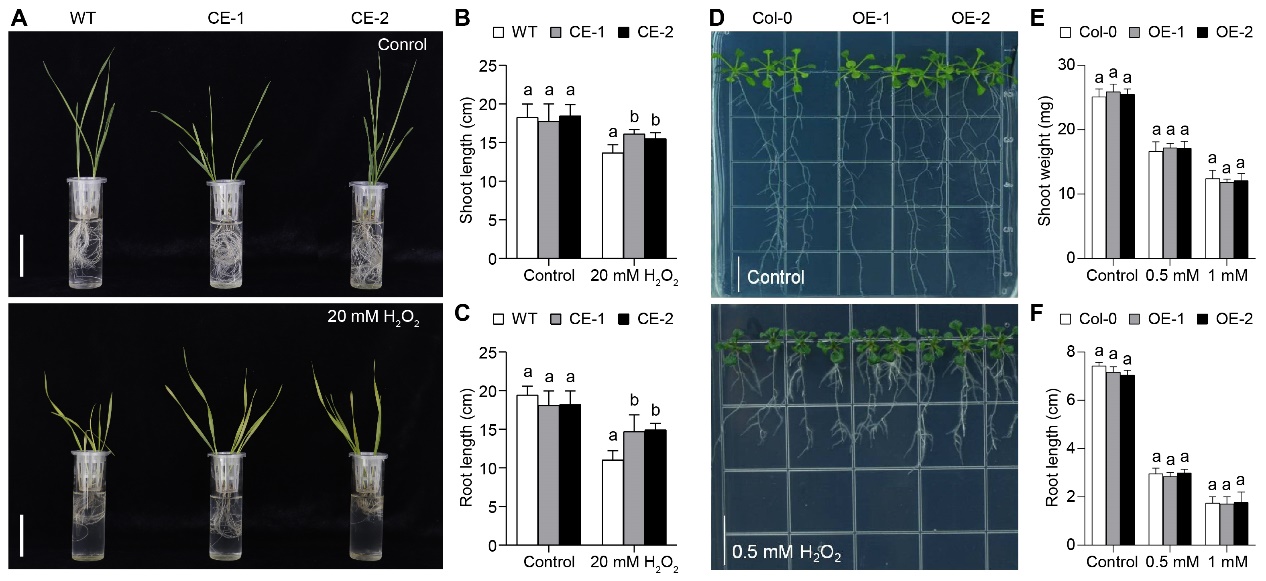
**

**Supplementary Figure 4**

**
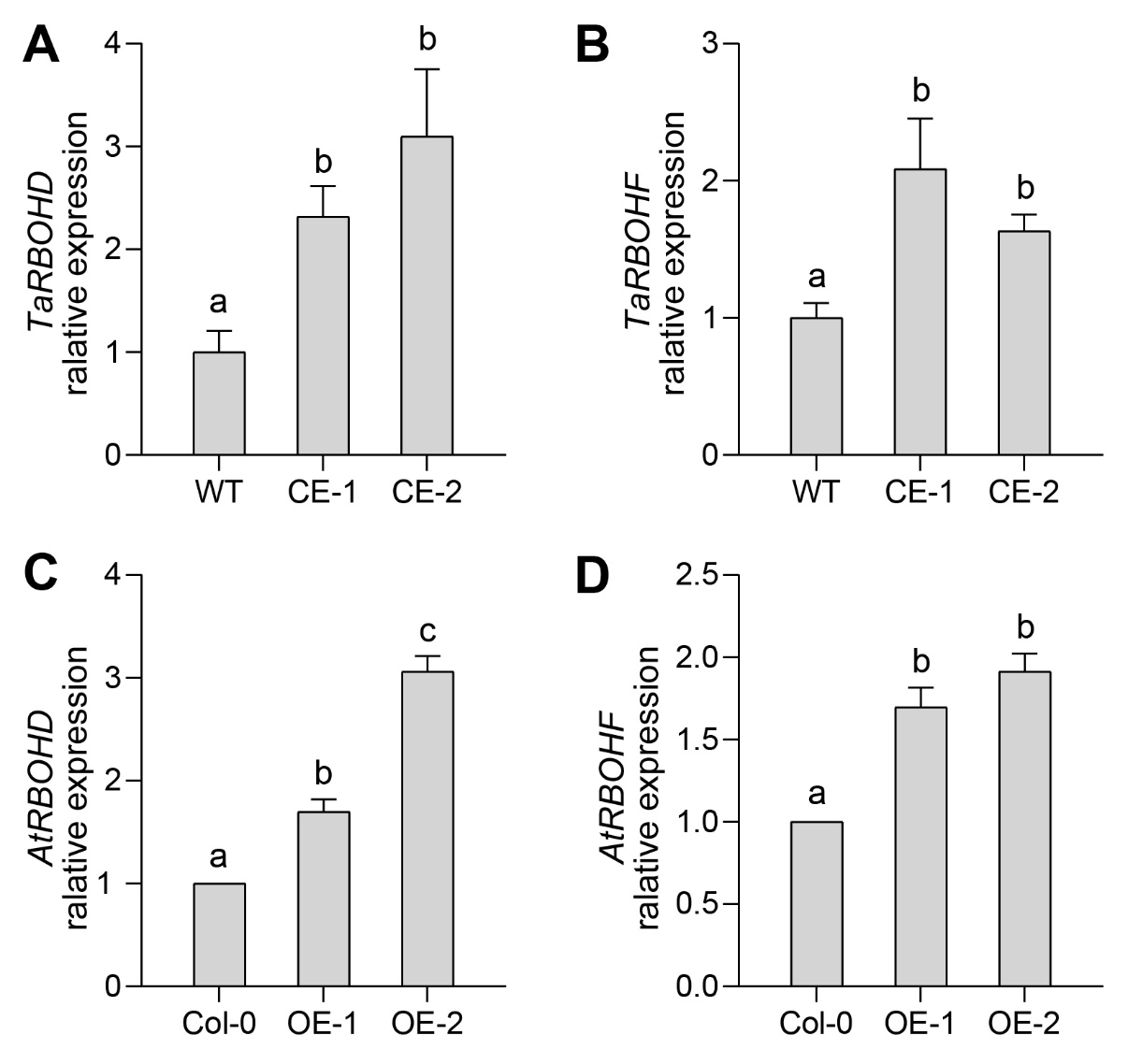
**

**Supplementary Figure 5**

**
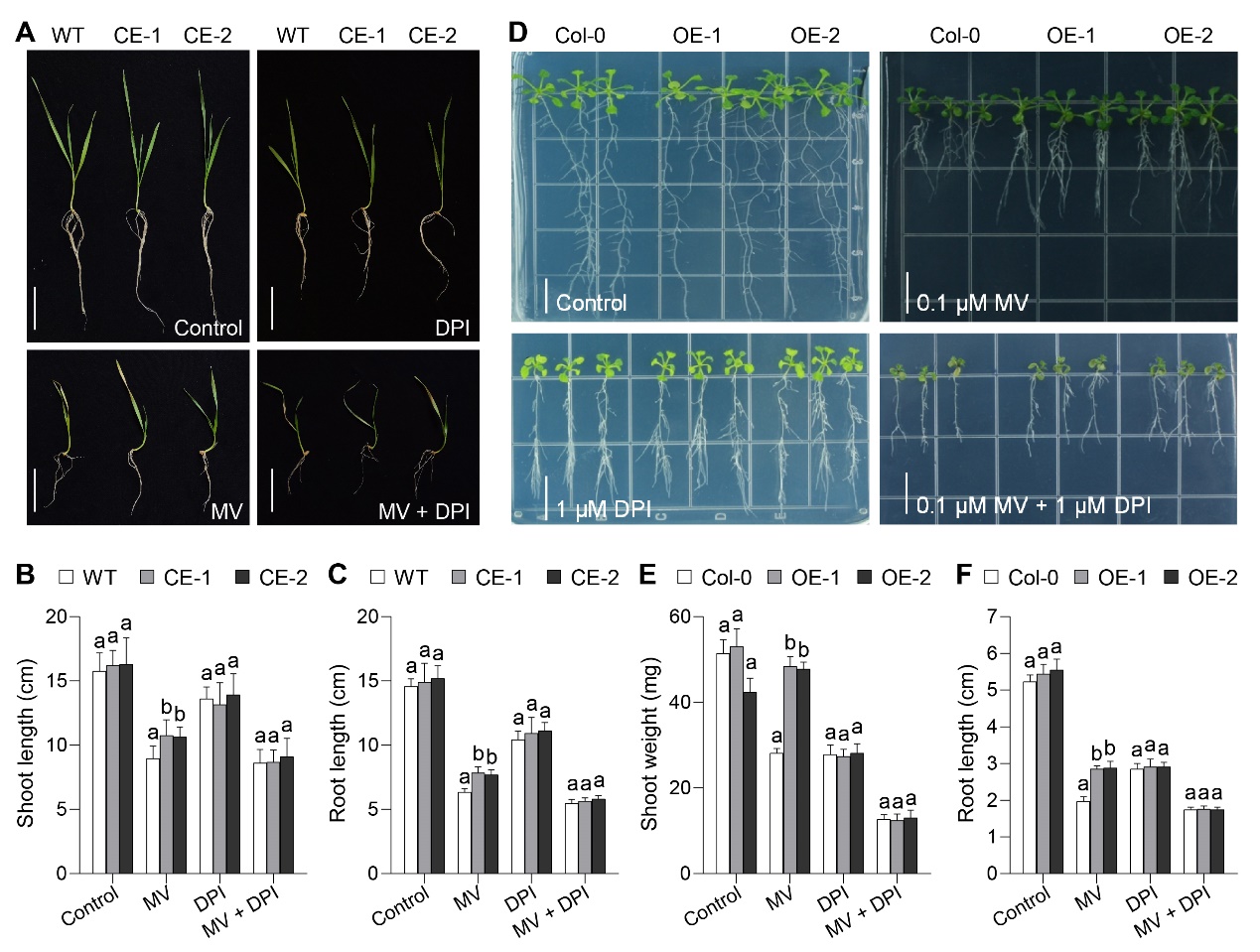
**

**Supplementary Figure 6**

**
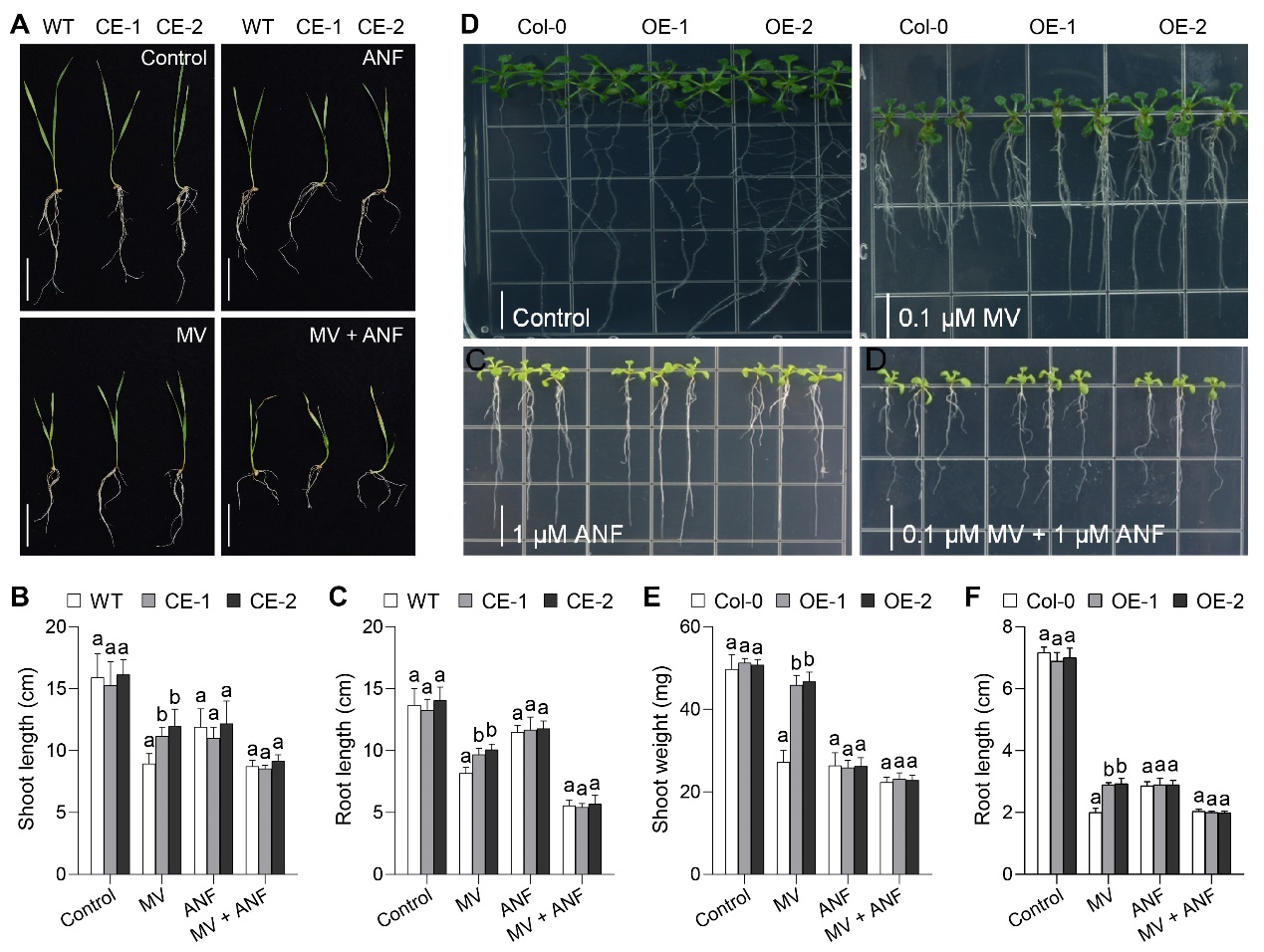
**

**Supplementary Figure 7**
